# Deep Brain Stimulation for Obsessive–Compulsive Disorder: A Long Term Naturalistic Follow Up Study in a Single Institution

**DOI:** 10.3389/fpsyt.2020.00055

**Published:** 2020-02-28

**Authors:** Marshall T. Holland, Nicholas T. Trapp, Laurie M. McCormick, Francis J. Jareczek, Mario Zanaty, Liesl N. Close, James Beeghly, Jeremy D.W. Greenlee

**Affiliations:** ^1^ Department of Neurosurgery, University of Iowa, Iowa City, IA, United States; ^2^ Department of Psychiatry, University of Iowa, Iowa City, IA, United States; ^3^ Rein Center: Emotional Health and Well-Being, Iowa City, IA, United States; ^4^ PennState Health, PennState University, Hershey, PA, United States

**Keywords:** neuromodulation, neurostimulation, neuropsychiatric disorder, deep brain stimulation, obsessive–compulsive disorder

## Abstract

**Introduction:**

Deep brain stimulation (DBS) is a proven, effective tool in the treatment of movement disorders. Expansion of indications for DBS into the realm of neuropsychiatric disorders, especially obsessive–compulsive disorder (OCD), has gained fervent interest, although data on appropriate clinical utilization remains limited.

**Methods:**

A retrospective, naturalistic study followed nine severely affected OCD patients (average YBOCs score before implantation 34.2 ± 2.5) treated with DBS of ventral capsule/ventral striatum, with average follow up of 54.8 months.

**Results:**

With chronic stimulation (years), a majority of the patients achieved significant benefits in obsessive–compulsive and depressive symptoms. Six patients experienced periods of OCD remission following implantation. Four of the six responders required more than 12 months to achieve response. Relief of major depressive symptoms occurred in four out of six patients with documented co-morbid depression. Settings required to achieve efficacy were higher than those typically utilized for movement disorders, necessitating increased impulse generator (IPG) battery demand. We found patients benefited from conversion to a rechargeable IPG to prevent serial operations for IPG replacement. For patients with rechargeable IPGs, the repetitive habit of recharging did not appear to aggravate or trigger new obsessive–compulsive behaviors or anxiety symptoms.

**Conclusions:**

Our study supports and builds upon other research suggesting that DBS for OCD in a real-world setting can be implemented successfully and provide long-term benefit for severely affected OCD patients. Optimal patient selection and DBS programming criteria are discussed. The use of rechargeable IPGs appears to be both cost effective and well-tolerated in this population.

## Introduction

Obsessive–compulsive disorder (OCD) is an anxiety disorder characterized by intrusive or persistent thoughts or urges (obsessions) that often lead to repetitive behaviors (compulsions) which cause significant distress or impairment in social, occupational, or other important areas of functioning. Compulsive behaviors are frequently performed in an attempt to counteract obsessions and relieve the anxiety associated with them (1). These behaviors can be time-consuming and create significant impairment in one or more areas of the patient’s life, interfering with his or her ability to live and function normally. The lifetime prevalence of this disorder is estimated to be 2–3% of the U.S. adult population (1–6).

The standard treatment for OCD involves a combination of medication and/or psychotherapy. Unfortunately, at least 10%, and as many as 20–40%, of patients suffer from treatment-refractory OCD (4, 7–14). Furthermore, major depressive disorder is a common comorbidity (15). This combination of low mood with obsessions and compulsive behaviors leads to a high rate of suicide, and it is estimated that up to one quarter of patients with OCD will attempt suicide (16).

Until recently, surgical options for treatment refractory OCD were limited to permanent ablative procedures such as anterior cingulotomy and anterior capsulotomy (17, 18). For example, a metaanalysis performed by Brown et al. showed a 37% reduction in Y-BOCs scores following cingulotomy and 57% for capsulotomy at 12 month post-procedure follow up (19). The field’s apparent hesitancy to pursue surgical intervention may be due in part to the controversial use of the highly indiscriminate prefrontal lobotomy to treat psychiatric disorders in the 1940’s and 1950’s, and this hesitancy has persisted despite the more accurate and discriminatory cingulotomy and capsulotomy, as they are also non-reversible and destructive lesioning procedures (20, 21). Advances in many areas of neurosurgery have led to the development of new, precise procedures such as radiosurgery and neurostimulation (22, 23). For example, deep brain stimulation (DBS) is a well-recognized and effective surgical intervention for the treatment of medically-refractory movement disorders (24, 25). With the increasing use of DBS for movement disorders, psychiatric effects of the stimulation have been observed, with benefits on both motor and non-motor symptoms (e.g. obsessions, compulsions, and mood) noted for patients with comorbid Parkinson’s disease and OCD (26, 27). These observations, combined with the knowledge of the clinical benefits from lesioning procedures in treating medically-refractory OCD, led to the development of DBS for OCD (28).

**Figure 1:**
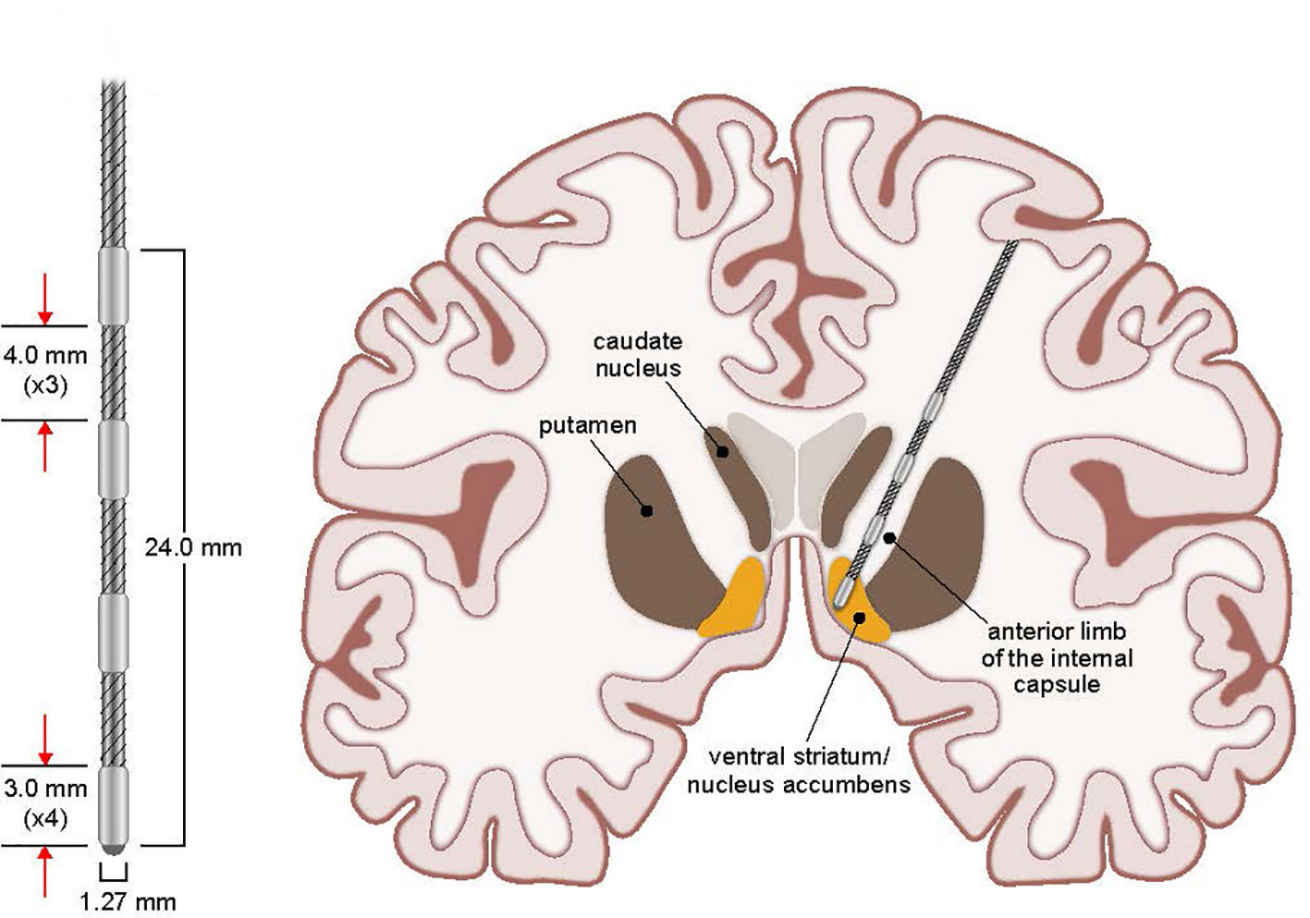
Stimulating Electrode and VC/VS Target. Coronal view schematic drawing of stimulating electrode placement and targeted stimulation of VC/VS for OCD. Additional details of the stimulating electrode measurements are presented enlarged at left.

DBS for psychiatric disorders is in its relative infancy. Nuttin et al. were the first to publish the use of DBS for the treatment of refractory OCD utilizing the same target as an anterior capsulotomy (29). Since then, multiple small studies have been published (9, 11, 29–37). An overview of the evidence is seen in the meta-analysis of 31 studies containing 116 subjects with targets including the anterior limb of the internal capsule, ventral capsule/ventral striatum (VC/VS), nucleus accumbens, inferior thalamic peduncle, subthalamic nucleus, and ventral caudate by Alonso et al. that demonstrated an average overall Yale–Brown Obsessive Compulsive Scale (YBOCS) score reduction of 45% and 60% responder rate (38). Additionally, specific evidence for VC/VS as a responsive target has been highlighted by several studies (33–36). This growing body of literature led the Federal Drug Administration (FDA) to approve a limited humanitarian device exemption to allow the use of DBS for the treatment of refractory OCD targeting the VC/VS (**Figure 1**). However, knowledge surrounding DBS for OCD is currently limited by several factors, including small sample sizes, lack of adequate controls, and use of multiple anatomic targets and stimulation parameters. An attempt to provide further evidence has been pursued; however, few long-term follow-up studies exist (39, 40).

As the application of DBS to neuropsychiatric illnesses such as OCD continues to evolve, we expect to learn more about the long-term course of neurostimulation for OCD. Additionally, we expect to improve methods for maximizing benefits and minimizing negative outcomes. This report describes the naturalistic study of patients with severe OCD that underwent DBS in a clinical setting outside of a research protocol, outlines the necessary steps to institute this procedure at an academic medical center, and offers a discussion of the associated lessons learned and unique findings which can provide insight into the use of DBS for OCD. We highlight the importance of an experienced surgical team, a multidisciplinary approach to treatment, and appropriate patient selection. Additionally, we examine financial implications of surgical treatment. Finally, we look critically at the longitudinal outcomes for our patient cohort, focusing on optimizing device settings and prognosticating response, drawing upon specific case examples to illustrate general themes.

## Methods

### Brief Study Overview and Approval

A retrospective chart review was performed to compile all known information pertaining to the DBS for OCD program at the University of Iowa (UI) from January 2010 to January 2018. The comprehensive, retrospective chart review included interviews with several providers from the multidisciplinary team involved in various aspects of the care continuum including referral, screening and selection of patients, DBS implantation, and post-implantation programming. This study was carried out with the recommendations of the University of Iowa Human Subjects Office. Written informed consent was obtained from all subjects for operative implantation. All subjects whose detailed clinical course are discussed in the vignettes gave written informed consent for this in accordance with the Declaration of Helsinki. The protocol was approved by the University of Iowa IRB-01 Biomedical Intuitional Review Board (IRB# 201703822).

### Patient Selection

A multidisciplinary steering committee was organized at the onset of this endeavor as part of the IRB application requirement to allow DBS for OCD to be provided at the University of Iowa Hospitals and Clinics (UIHC) under the FDA humanitarian device exemption. The committee met regularly whenever a patient was referred for possible DBS for OCD treatment. This committee included members from the Departments of Psychiatry, Neurosurgery, and Neuropsychology. Potential patients were identified by Department of Psychiatry physicians at the UIHC. Those patients were referred to the steering committee for formal evaluation for potential DBS implantation. The committee would review the patient’s complete medical and psychiatric history and required: 1) an unambiguous DSM-IV (later, DSM-5) diagnosis of OCD, 2) evidence of unresponsiveness to all prior indicated therapies, and 3) exhaustion of all other possible effective therapies. In detail, the committee came up with the following inclusion criteria: 1) Documented OCD symptomatology of 5 years or longer; 2) OCD rated as severe (i.e., YBOCS score >30 in most cases), 3) Failure of at least three adequate trials of selective serotonin reuptake inhibitors (SSRIs), 4) Failure of cognitive behavioral therapy or other OCD-targeted psychotherapy, and 5) Age of 18 years or greater. Exclusion criteria included 1) Evidence of hoarding disorder, 2) Evidence of serious comorbid personality disorder pathology, 3) Evidence of active substance abuse issues, 4) Pregnancy, 5) Serious neurologic illness such as dementia (although seizure disorders were allowed if adequately controlled), 6) Serious bleeding disorder or use of chronic blood thinners, 7) Requirement of regular MRI monitoring for another disorder, and/or 8) Other contraindication to undergoing neurosurgery (see **Table 1**). Although some of these requirements were based on previous prognostic evidence (e.g., documentation of poor response in hoarding disorder subtype of OCD) or practicality (e.g., patients with dementia), others were based on clinical judgment and suspicion of poor candidacy (e.g., excluding patients with serious personality or substance abuse pathology due to concern for self-harm or difficulty assessing efficacy with severe comorbidities) (41).

**Table 1:** Inclusion and exclusion criteria used in patient selection.

**Inclusion:** Diagnosis of OCD for duration of 5+ years OCD rated as severe (YBOCS score 30+) Failure of three or more serotonergic medications (SSRIs or clomipramine) Failure of an adequate trial of cognitive behavioral therapy or other form of OCD-targeted psychotherapy 18 years of age or older
**Exclusion:** Hoarding disorder diagnosis Serious comorbid personality disorder pathology Serious substance abuse issues Neurosurgical contraindication Previous surgery to destroy the DBS brain region target Pregnancy Serious neurologic disorder such as dementia (controlled epilepsy permitted) Bleeding disorder or requirement for chronic blood thinner use Requirement of routine MRI monitoring for another condition

If the patient satisfied the inclusion criteria and was medically fit for surgical intervention, he or she was declared a candidate for DBS. Attention would then be turned to insurance approval and reimbursement capabilities. The most common reasons for exclusion were lack of a clear OCD diagnosis or significant psychiatric comorbidity such as a history of self-harm or picking behaviors. Other reasons for exclusion were inadequate pharmacologic or psychotherapeutic trials, poor surgical candidacy, and/or inability to secure a reimbursement plan.

### Operative Technique

The implantation of the DBS system was typically performed in a staged fashion using techniques consistent with DBS implantations at UIHC for other indications. This involved separate surgeries for cranial electrode implantation (Stage I) and impulse generator implantation (Stage II). Prior to first surgery (Stage I, implantation of brain electrodes), patients underwent a volumetric MRI scan (1 mm slice thickness) that was loaded into a computer workstation (Stealth Framelink, Medtronic, Inc., Minneapolis, MN). The bilateral VC/VS were identified and direct targeting was performed. Target placement and electrode details are shown in **Figure 1**. Appropriate entry points and trajectories were chosen to avoid crossing sulci, major vessels, and ventricles with the lead traversing down the anterior limb of the internal capsule. Frame-based stereotaxy (CRW, Integra, Inc.) was used *via* co-registration of the MRI with a volumetric CT scan obtained the morning of surgery after stereotactic frame placement. Surgery commenced with the patient awake and under local and monitored anesthesia with intermittent, short-acting anxiolytics and analgesics (e.g., propofol, dexmedetomidine). After both DBS leads (3391, Medtronic, Inc., Minneapolis, MN) were implanted *via* pre-coronal burr holes, the conscious sedation was stopped for behavioral testing at various unilateral stimulation settings. The primary goal of this testing was to evaluate a variety of voltages and pulse widths while concurrently monitoring for evidence of acute therapeutic benefit or side effects. In addition to the operating and anesthesia teams, others present during the operation included medical device company representatives experienced in OCD DBS surgery and the patient’s psychiatrist. The psychiatrist or medical device representative would perform intraoperative testing for evidence of acute changes in mood or OCD symptoms, including smile or laughter induction, changes in anxiety, happiness, or energy level. Validity of the effects was often confirmed by performing testing with the patient blinded to device parameters/on-off status. A subset of patients experienced a euphoric feeling upon the initiation of acute stimulation at the target unilaterally. While this observation has been noted in prior literature of DBS at this location, the long term clinical importance of this is unclear (42). The newly implanted leads were then coiled unilaterally under the scalp and the wounds closed. All patients were monitored in the hospital overnight and typically discharged home the following day. No transient “lesion” effects were noted by patients or their families or evident in the medical records for any patient in the days following lead implantation surgery.

Stage II surgery was performed 10 to 14 days after completion of the first surgery. This procedure involved placement of the impulse generator (Soletra, Kinetra, or Activa PC, Medtronic, Inc.) in the chest and connecting the previously placed cranial electrodes to the generator *via* extension cables. This operation was conducted under general anesthesia as an outpatient procedure.

It was not our clinical practice to routinely obtain post-operative imaging to evaluate lead position in our implanted patients, nor did we image those patients (e.g. #’s 6, 7, 9) that were implanted at outside institutions. In using frame-based stereotaxy and current Microdrive systems and based on our experience with hundreds of similar DBS implant methods in movement disorder patients, we estimate the variation in lead position from intended to actual target is ~ 2 mm. The only patient we implanted that was classified as a non-responder (#8) did have imaging sufficient to evaluate lead locations, and both leads were confirmed to be in the anterior limb of the internal capsule and within the published range of direct targeting coordinates for VC/VS.

### Clinical Measures and Follow-Up

Follow-up and DBS device programming by the treating psychiatrist began approximately two to four weeks after implantation of the impulse generator(s). This involved testing various parameter settings and monitoring for acute physical and psychiatric symptom changes. When possible, initial programming was directed by the results of intraoperative testing, with the ultimate goal of the programming physician to maximize benefits and minimize side effects of the stimulation. In some cases, however, intraoperative testing notes provided minimal guidance, leaving the treating psychiatrist to adjust parameters based on previous programming experience and/or side effect burden. In general, adjustments were made to the voltage initially, with additional changes of pulse width, frequency, and contact configuration if thought to be of potential benefit. Patients initially followed up weekly with their psychiatrist, with small incremental adjustments made to the DBS device until a tolerable setting was achieved that was perceived to be providing benefit. Follow-up visits were then gradually spaced out based on patient’s response and comfort level, with some patients extending their follow-up interval to 6 months or more.

Follow-up visits included a variety of rating scales to measure symptoms and monitor response; these included the YBOCS, Hamilton Depression Rating Scale (HAM-D), Montgomery-Asberg Depression Rating Scale (MADRS), Quick Inventory of Depressive Symptomatology (QIDS), Inventory of Depressive Symptomatology (IDS-30), Beck Depression Inventory (BDI), Beck Anxiety Inventory (BAI), Generalized Anxiety Disorder 7-item Scale (GAD7), Montreal Cognitive Assessment (MOCA), and a Global Assessment of Functioning (GAF) score. Consistent with prior literature, a positive response to treatment was defined as 35% improvement in YBOCS scale score and remission was defined as a total YBOCS score < 12 (41). Depression remission was defined as HAMD-21 < 8, PHQ9 < 5, or MADRS < 8 (43). Scales were employed variably based on physician discretion, and thus not every scale was obtained for each follow-up visit.

### Cost Analysis

Hardware charges were obtained *via* chart review. Impulse generators were categorized as either non-rechargeable or rechargeable. All figures were adjusted for inflation to January 2018 U.S. Dollars for comparability utilizing the U.S. Bureau of Labor Statistics consumer price index inflation calculator to the nearest dollar. Hardware charges, rather than collections, were used to perform calculations to improve comparability of the figures and avoid any differences in insurer payment schedules.

## Results

### Patient Demographics

The UI has cared for a total of 9 patients with an implanted DBS system for treatment of OCD from January 2010 to January 2018. Six of these patients had their initial DBS implantation procedure performed at our institution. One patient had a combined procedure in which Stage I and II were performed in one operation. The average age of patients at implantation was 40.2 ± 13.6 years. There were five males and four females.

Patients had undergone an average of 13.2 trials of medications for their OCD prior to implantation; this number includes augmentation agents such as benzodiazepines and antipsychotics. Although failure of augmentation agents was not part of the inclusion criteria for patient selection, all nine of the patients had failed either augmentation with one or more antipsychotics (three of nine), clomipramine (one of nine), or both (five of nine). All had failed a trial of cognitive-behavioral therapy, primarily exposure and response prevention therapy, or some form of psychotherapy targeted to address the OCD symptoms and behaviors and deemed adequate or intolerable by the evaluation team. Four of nine had failed electroconvulsive therapy, and one had failed off-label repetitive transcranial magnetic stimulation. All nine patients remained on pharmacotherapy in addition to receiving DBS therapy.

Many patients had additional DSM-IV diagnoses as outlined in **Table 2**; the most common comorbidity was major depressive disorder (seven of nine), although other comorbidities included generalized anxiety disorder, eating disorder, personality disorder, tic disorder, panic disorder, and psychosis not otherwise specified. The average length of time in the OR for patients undergoing Stage I surgery was 245 ± 50 min.

**Table 2:** Patient characteristics.

Patient	Age at Stage 1 surgery (5 year ranges)	Baseline YBOCS	# of Prior Medication Trials*	Age of OCD Onset (5 year ranges)	Additional Psychiatric Diagnosis
Patient 1	26–30	31	9	16–20	None
Patient 2	46–50	32	19	10–15	MDD, GAD; Prior cingulotomy
Patient 3	26–30	35	9	10–15	MDD, History of hoarding
Patient 4	46–50	37	14	21–25	Anorexia nervosa, tic disorder, depression NOS
Patient 5	61–65	34	10	31–35	MDD, GAD, panic disorder
Patient 6	36–40	36	4	21–25	MDD
Patient 7	36–40	31	16	11–15	MDD, GAD
Patient 8	16–20	34	19	11–15	MDD, GAD, psychosis NOS, Tourette’s
Patient 9	40–45	38	19	11–15	MDD

MDD, major depressive disorder; GAD, general anxiety disorder; NOS, not otherwise specified.

*Prior medication trials total number includes all primary and augmenting psychotropic agents, including those of adequate dose-duration and those the patient stopped early for intolerance or other reasons.

### Clinical Outcomes

**Figure 2:**
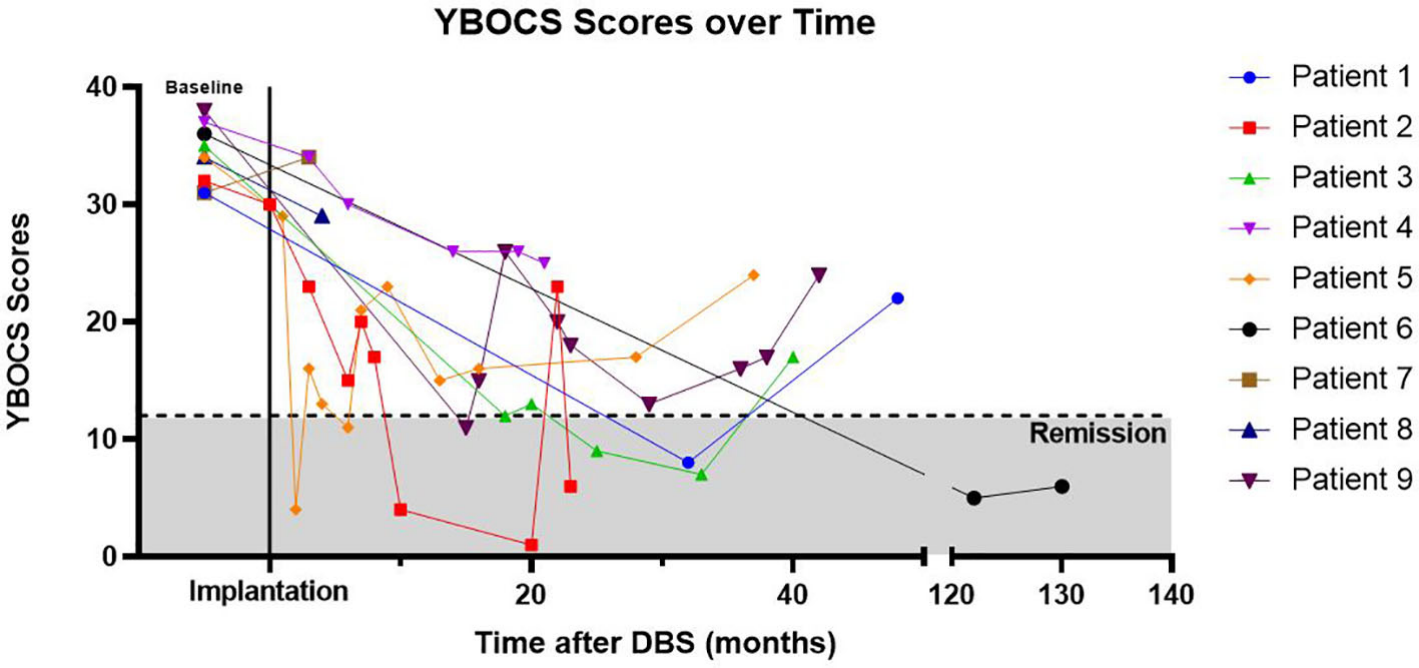
YBOCs Scores Over Time. Yale–Brown Obsessive Compulsive Scale (YBOCS) scores over time, with time T = 0 referring to baseline YBOCS score immediately prior to DBS implantation. Cumulative correlation coefficient r = -0.480. Patient 6 had significantly longer follow-up than other patients and line is abbreviated to aid with visualization (note break in the x-axis denotes change in time-scale).

The most consistently obtained measure in this group of patients was the YBOCS. This was used as our primary outcome measure to evaluate DBS effectiveness (**Table 3**). The average initial YBOCS score was 34.2 ± 2.5 (range 31–38). Patients were followed on average for 54.8 months (range 5–130), with eight of nine being followed in clinic for 22 months or longer. Based on YBOCS scores, six of the nine patients achieved OCD remission (defined as a YBOCS score <12) at some point during the course of their treatment while three subjects were designated ‘non-responders’ (31). Individual patient YBOCS scores over time are presented in **Figure 2**. The average time to response was 32.5 months for responders, although this was largely driven by an outlier with a significantly prolonged time to response; with the outlier removed, the average time to response was 14.6 months (range 2 to 32 months). Among the six responders, two were in remission by 12 months, and four reached remission by 24 months. Of the three patients that did not achieve remission, none achieved response by YBOCS criteria. This finding suggests that in the group of patients who received benefit from DBS treatment, the effects were robust; in the non-responder group, there was almost no appreciable clinical effect of stimulation. Few of our patients fell into the “middle ground” with a partial response without remission of symptoms. The exception to this was patient 4, who had a 32% improvement in YBOCS, just below the defined cut-off (35%) for being a responder. Interestingly, several patients showed delayed OCD relapse (**Figure 2**, **Table 3**). At the final follow-up timepoint in this review, only two of nine remained in OCD remission and another two subjects met criteria for sustained response.

**Table 3:** OCD outcomes for all patients.

Patient	Baseline YBOCS	Best YBOCS after DBS	Time to Best YBOCS (mos)	Best Response Type	Last YBOCS	Time to Last Follow Up (mos)	Comment
Patient 1	31	8	32	Remission	22	48	DBS removed
Patient 2	32	1	20	Remission (+MDD Remission)	6	61	Hx of prior cingulotomy
Patient 3	35	7	33	Remission	17	63	Hx of hoarding
Patient 4	37	25	21	32% Improved	25	72	
Patient 5	34	4	2	Remission (+MDD Remission)	24	50	Unilateral left lead stimulation only
Patient 6	36	5	122	Remission	6	130	Non-UI implant
Patient 7	31	34	n/a	Non-responder	34	5	Non-UI implant
Patient 8	34	29	4	Non-responder (+MDD Remission)	29	22	
Patient 9	38	11	15	Remission (+MDD Remission)	24	42	Non-UI implant

MDD, major depressive disorder; UI, University of Iowa.

In regards to secondary outcome measures, the most commonly obtained measure was that of depressive symptoms. Seven of the nine patients in this study carried diagnoses of major depressive disorder (MDD), and an eighth had depression not otherwise specified by DSM-IV criteria. Of the seven with MDD, six were documented as currently experiencing a major depressive episode by clinician evaluation, rating scale scoring, or both. Of the six with active depressive symptoms, four patients experienced remission of depressive symptoms following activation of the DBS system.

DBS was well tolerated with few side effects. There was one case of hypomania that occurred after rapid up-titration of a patient’s device (See “Patient Perspective, Responder” below). Other noted side effects included: vertigo/dizziness, racing thoughts, euphoria, restlessness, decreased sleep, anxiety, urge to urinate, abnormal smells, and abnormal movements/seizure-like activity. These side effects were almost always transient or setting-specific and resolved with device parameter adjustments. One patient interestingly was noted by the treating psychiatrist to have an immediate improvement in visual acuity with stimulation, as well as acute beneficial changes in range of speech intonations and affective brightness. Although it was not systematically studied, it is noteworthy that seven of nine patients were able to reduce pharmacotherapy after DBS implantation, qualified as either a dose reduction of prescribed psychotropic medication or a discontinuation of a previously prescribed psychotropic.

### Individual Case Vignettes

#### Responder

The first case we present is a patient in his 40s who had been experiencing OCD symptoms since age 12 (Patient 2). These symptoms included hyperreligious obsessions, obsessions of self-doubt, and obsessive thoughts related to body appearance and weight. The patient had been hospitalized numerous times for his symptoms, had several suicide attempts, and was so impaired that the patient required assisted living at various points. The patient had failed numerous medication trials including SSRIs, SNRIs, antipsychotics, TCAs, MAOIs, benzodiazepines, and several other psychotropic augmenting agents. Moreover, the patient had failed a trial of ECT. The patient ultimately was diagnosed with comorbid eating disorder, major depressive disorder, and generalized anxiety disorder. The patient underwent a cingulotomy for OCD symptoms in his 20s, which provided limited benefit at best: per records, the patient was hospitalized less frequently and had less frequent suicide attempts following the cingulotomy but was still disabled and largely unable to function.

After DBS implantation, the DBS parameters were set to 4.2 volts, 210 μs pulse width, and 135Hz frequency bilaterally at his first outpatient programming session. Although the patient did not report any subjective changes during the programming visit, the provider noted the patient to be smiling more with brighter affect once the amplitude was increased above 2.0 volts. Within 2 h, the patient began to make out-of-character grandiose and hypersexual comments. He began to engage in disinhibited speech and behaviors that concerned his wife and children. The patient returned to clinic for programming adjustments, ultimately having the voltage decreased to 0.5 volts bilaterally. Within minutes to hours, the hypomania symptoms resolved and the patient was able to undergo more conservative programming and gradual up-titration of voltage. In the long term, the patient had a robust response to the DBS, with YBOCS scores decreasing from 32 to 4 over the following 10 months. Depressive symptoms also went into remission. The patient’s symptoms have remained in remission for the past 7 years, with a single exception. Approximately 2 years after initial device implantation, the battery had reached the end of its usable life. Following the battery change, the patient’s device voltage was set approximately 25% lower than the prior voltage. The patient experienced a rapid decline in his symptom control within a week, returning to the point of being completely disabled (YBOCS 23). Once the discrepancy was discovered and the device was reset to his standard settings, the OCD symptoms rapidly resolved and were in remission within one month. The patient has remained in remission since then for the past 5 years, and is in the process of endeavoring to open his own business.

#### Non-Responder

One notable DBS non-responder in our patient series suffered significant comorbidities that were outside the mood and anxiety disorder spectrum. One of the two patients had anorexia nervosa and a tic disorder, while the other had psychosis not otherwise specified, Tourette’s syndrome, and other comorbid neurological disease. Significant comorbidities such as these add a level of complexity to the cases which often worsens outcomes of all the disorders, irrespective of the proposed therapy of choice. For example, the patient with comorbid anorexia had several OCD symptoms that complicated the anorexia symptoms, such as obsessive anxiety about eating foods the patient deemed “impure,” or mixing certain foods together. The patient with neurological comorbidity and psychosis had significant behavioral problems that limited the patient’s ability to participate in any beneficial form of psychotherapeutic intervention. These cases highlight the fact that neuropsychiatric comorbidity with OCD is common and increases the likelihood of treatment failure (44).

It was determined that DBS was indicated and pursued as the patient was severely ill and had failed all other treatment options. Despite failing to meet the response or remission criteria as defined by YBOCS score, this patient had significant clinical improvement. Prior to DBS treatment the patient with comorbid anorexia was hospitalized every few months to years; following DBS implantation, the patient required one single hospitalization in the subsequent 6 year period. The patient’s YBOCS scores improved 32%, just failing to meet the threshold for treatment response. The patient subjectively reported improvement from the DBS despite continued impairment from OCD and anorexia nervosa symptoms. Similarly, the patient with comorbid epilepsy and psychosis also had subjective improvements (YBOCS scores improved 15%) which translated into the patient’s ability to graduate from high school and pursue gainful employment, whereas at baseline the patient required constant monitoring and institutionalization. These case reports suggest that it may be worthwhile to re-evaluate the current literature’s definition of treatment response in OCD based on YBOCS scores alone. To more adequately characterize treatment effect, additional evaluations including patient-reported outcomes and quality of life measures may be useful.

### Device Settings

Device settings for responders were: average voltage 4.57 V (range 2.5–8.0 V), frequency of 135 Hz (5 of 6) and 90 Hz (1 of 6), and pulse widths of 210 μs (3 of 6), 180 μs (1 of 6), 120 μs (1 of 6), and 90 μs (1 of 6). All of the responders had unipolar device settings with the case serving as the anode and the deepest (i.e. numbers 0 or 1) contacts serving as the cathode. These settings did not differ significantly from the maximum settings employed for the 3 non-responders (mean voltage 5.0 V; frequencies of 140, 130, and 90 Hz; pulse widths of 300, 210, and 180 μs; case served as anode for two of three patients with one contact as cathode, while third patient had bipolar settings with contact 3 anode and contact 0 cathode). Six of the nine patients followed were eventually implanted with rechargeable batteries. Eight of nine patients utilized bilateral stimulation settings, whereas one patient used left unilateral stimulation. The optimal device settings for each of the six responders are outlined in **Table 4** below.

**Table 4:** DBS device settings associated wit hgretaets symptomatic improvement for individual responders.

Patient	Voltage	PW (ms)	Freq (Hz)	Contacts
Patient 1	5.2L/5.8R	180B	135B	C+/1-
Patient 2	3.8B	210B	135B	C+/1-
Patient 3	5.1B	120B	135B	C+/0-1-
Patient 5	2.5L/0.0R	210B	90B	C+/0-
Patient 6	4.0L/4.5R	210B	135B	C+/0-1-
Patient 9	8.0L/7.0R	90B	135B	C+/1- L C+/0- R

L, left; R, right; B, bilateral; C, case (monopolar setting).

### Cost Analysis

**Figure 3:**
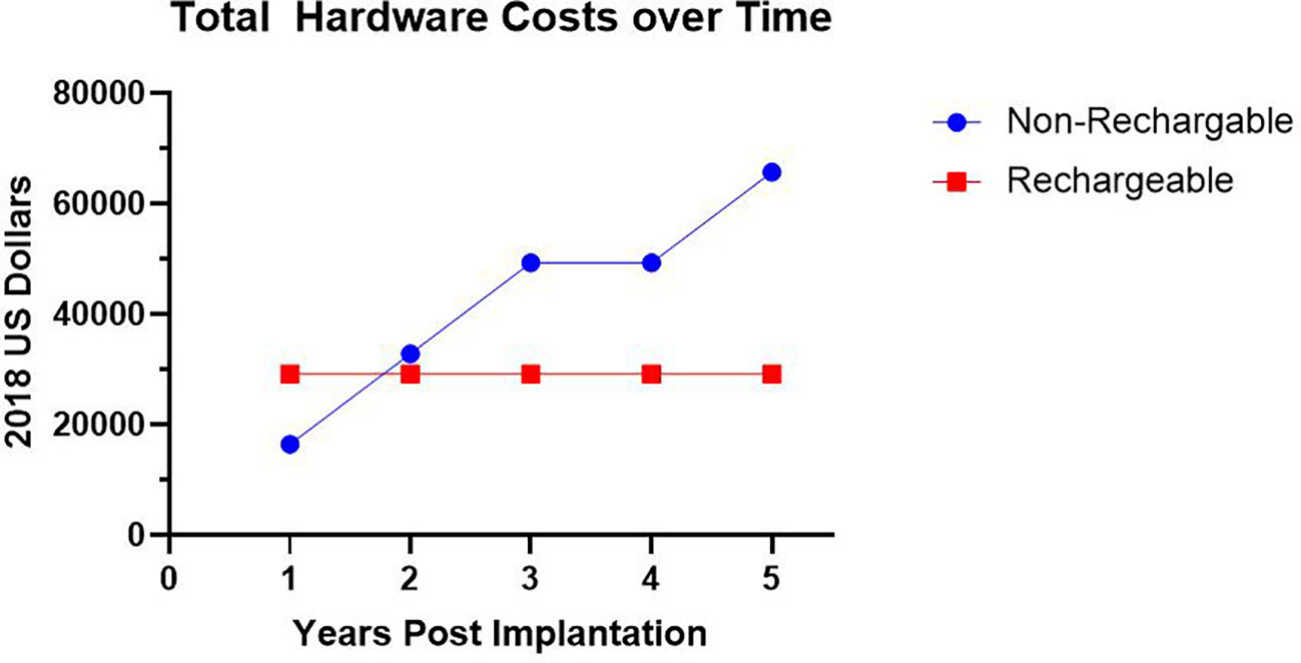
Hardware Costs Over Time. Average total hardware costs in 2018 US Dollars utilizing a non-rechargeable system requiring impulse generator replacement versus rechargeable system over time.

Seven patients had procedures at our institution with cost information available in the medical record. Six of these patients had their initial implantation performed at our institution. The average cost for initial implantation hardware was $27,035 ± $3,623. For these six participants there was an average cost per month usage until need of initial battery replacement of $1,878 ± $1,019. For the six patients that have charge information for impulse generator changes, we found the average cost per month of use for a non-rechargeable impulse generator to be $1,517 ± $870. For rechargeable impulse generators, we found the average cost per month to be $654 ± $219. We found that on average, a non-rechargeable battery required replacement every 1.4 years with an average hardware cost of $16,432 ± $9,163. Despite the higher initial hardware costs of a rechargeable impulse generator, the rechargeable system became more cost effective by 1.6 years following initial implantation, primarily due to the mitigated cost of battery replacement (**Figure 3**).

## Discussion

### Clinical Outcomes

In our series of 9 patients, we found that treatment with DBS resulted in an overall OCD remission rate, defined by YBOCs scores <12, of 67%.Our rate is consistent with prior literature on long-term follow-up of comparable patients with VC/VS DBS (15, 45). The average time to clinical response was 32.5 months. Time to response for the 6 individuals were as follows: at 12 months after surgery 2 had remitted, at 24 months 2 more had remitted. This gradual response over months to years is also consistent with prior reports (7). For example, in a similar size sample of OCD patients with VC/VS DBS stimulation followed for 36 months, Greenberg et al. showed that only one of 10 patients had responded at 1 month, and that number increased to 4 of 8 by 36 months (46). The peak mean improvement in YBOCS scores in that study was not achieved until 24 months post-implantation, with the slope of symptom decrease not stabilizing until approximately 12 months. This prolonged time to maximum treatment response followed by long-term sustained response is a phenomenon also commonly seen in the use of vagal nerve stimulation and DBS for treatment-resistant depression, possibly suggesting a similar underlying mechanism of action for these two modalities that portends a positive long-term clinical benefit (47–49).

While 6 of 9 patients met remission criteria following implantation, only 2 of 9 remained in remission from OCD symptoms, and an additional two patients met criteria for sustained response at the time of their most recent follow up appointment. Thus, although the literature suggests that the benefits from DBS may not occur for weeks to months after implantation, the lack of sustained remission for some patients in our study suggests that a prolonged response cannot be assumed, even for those who have an initial therapeutic gain. Additionally, there is prior literature that has noted a general trend of increasing voltage used over time, presumably to maintain therapeutic efficacy (50). As our study was merely observational and not designed to rigorously control other aspects of the patient’s care and environment, it is possible that uncontrolled events and circumstances unrelated to DBS contributed to disease relapse; for example, patients continued to have medication adjustments made by their psychiatrists during the observational period, and many continued to experience significant psychosocial stressors (51). Further complicating interpretation of clinical impact, we found physician documentation was occasionally incongruent with objective measures, the most common scenario being patients or physicians reporting significant improvements despite minimal change in the patient’s YBOCs score.

These results highlight the difficulty in assessing for response in a “real-world” setting. This difficulty takes two primary forms: 1) difficulty delineating true DBS effects from a placebo effect or the impact of other therapies, life events, and situational stressors; and 2) difficulty assessing true quality of life changes using standard rating scales. Despite these challenges, the fact that six of our nine patients had such a robust response after failing numerous standard medication and psychotherapy treatment trials is promising. Additionally, given that this patient population has a notoriously low placebo response, psychotropic medications were able to be reduced over time in the majority of patients (7 of 9) in this chronically ill sample, and the symptomatic improvements of our patients were time-correlated with the implantation of the DBS device, our findings strongly suggest that DBS treatment was driving much of the benefit they experienced (52).

### Learning From Case Vignettes

#### Responder

A responder patient’s case (Patient 2) emphasizes a few important points about DBS as a therapy for OCD. First, the pathophysiology of OCD is complex. High frequency stimulation of the VC/VS target has been purported to decrease hyperactivity in the orbitofrontal cortex, a metabolic phenomenon often associated with OCD although the precise mechanism underlying the effect is largely unknown (53–55). Second, although cingulotomy remains an established, albeit uncommon, neurosurgical treatment for OCD, it failed to have an effect in this patient, who then responded robustly to DBS targeted to a different anatomic location. As OCD is likely related to a corticostriatothalamocortical circuit dysfunction, the underlying circuit may not function in the same manner in all individuals, suggesting OCD may be a heterogeneous illness (56–59). This heterogeneity is also evidenced by the fact that OCD symptoms manifest in various forms. For example, this patient’s symptoms were primarily related to self-doubt, pseudo-eating disorder symptoms, and hyperreligious thinking. Some evidence suggests that different OCD subtypes may respond differently to DBS. For instance, it is generally accepted that hoarding disorder subtypes of OCD respond poorly to DBS (41). Interestingly, one meta-analysis showed that sexual and hyperreligious obsessions and compulsions were associated with significantly better response to DBS when compared to other subtypes of OCD (38). This patient’s case supports this finding.

A second theory for Patient 2’s DBS response following a failed cingulotomy is that the effects of DBS are mechanistically distinct from those of a lesion. Some authors theorize that DBS acts by stimulating orthodromic and antidromic corticostriatal fibers to modulate involved brain regions including the orbitofrontal cortex, anterior cingulate cortex, and the thalamus (10). Unlike a lesion, which disrupts all information transfer through the affected location, evidence suggests DBS can interrupt pathologic neural activity in the corticostriatothalamocortical circuit while allowing continued normal information transfer between regions (45).

Finally, this patient’s case is consistent with prior reported cases in the sense that the response was gradual over the course of several months, and then sustained for years, with significant improvements in the patient’s quality of life, including fewer hospitalizations, less suicidal ideation and fewer suicide attempts, improved psychosocial and occupational functioning, and less depressive and OCD symptom burden. The patient experienced rapid recrudescence of his symptoms with an inadequate DBS setting, and then improved with resetting the device, at a rate of improvement that was faster than the initial resolution of symptoms. The finding that symptoms improve more rapidly when a device is reprogrammed is interesting and may imply that the device was already “priming” his circuit to respond more rapidly to an increased voltage, or could suggest that some element of sustained benefit from the long-term use of DBS was present—some studies suggest DBS-induced neurogenesis or alterations in plasticity can persist even without adequate DBS settings (7). Further exploration of these types of observations are critical for the generation of novel insights about how DBS effects change in OCD brain pathophysiology, and more generally how the brain integrates neurostimulation.

#### Non-Responder

Given that we currently have no strong predictors of response or non-response, in concert with the challenges of consistently defining what determines a clinically-significant response, there is much to be learned from those OCD patients who did not respond robustly to DBS. Herein, we present a interesting non-responder case. The patient with anorexia nervosa (Patient 4) was treated in the hope that the anorexia would improve with successful treatment of the OCD, given that many of the obsessions and compulsions were food-related. Indeed, at least one case report has demonstrated efficacy of VC/VS DBS for OCD with comorbid anorexia nervosa, with concurrent improvements in anorexia symptoms (60). Nonetheless, the patient had poor response to DBS treatment. Thus, significant psychiatric comorbidity is one possible risk factor for the failure of this therapy. Further support for this theory comes from a second non-responder in our cohort with a comorbid psychosis NOS diagnosis. Another risk factor for failed therapy is age of onset of the disease. Two of our three non-responders had onset prior to age 15. At least one meta-analysis has found that later onset of illness is associated with better DBS response, suggesting a poorer prognosis for these patients (38).

Additionally, it is likely that unintended, diffuse neurologic damage will decrease the likelihood of response to any therapy, including DBS; in two of our non-responder cases, neurologic damage was suspected although not clearly evident on imaging (one patient with possible anoxic brain injury, the other with multiple episodes of severe caloric restriction and malnutrition from her eating disorder). Further research would need to elucidate the exact role these types of neurologic insults may play in predicting response to DBS for OCD, or for any other neuropsychiatric indication. Finally, it is interesting that two of our non-responders have a tic disorder. Current evidence would not necessarily suggest this to be a poor prognostic factor and in fact there are cases studies of patients with both OCD and Tourette’s responding to DBS (61, 62). Though pursuing DBS was a reasonable treatment choice considering the severity of impairment in this patient cohort, three patients failed to respond based on the defined criteria. This highlights the current sparseness of evidence and the need for diligent observation by providers to direct future treatment.

Identifying good and poor DBS candidates prior to implantation can be challenging and should be the focus of future research. Especially considering the not-insignificant risk associated with DBS implantation, the substantial costs associated with the procedure, and the additional lifestyle changes required of the patient once implanted (e.g., maintaining and monitoring the battery, adjusting to the cosmetic changes of small bumps on the head and a bulge in the chest for the battery pack, etc.), a better understanding of prognostic factors and biomarkers of response is imperative to the ongoing use of DBS for OCD and future expansion of indications to other neuropsychiatric disorders.

### Optimizing Device Settings

Another area of great need in the field of DBS for OCD is the efficient and effective determination of optimal DBS device settings. Although limited by the retrospective nature of this review, we were able to identify the device settings for each of our six responders at which they experienced their optimal time-correlated response (**Table 3**). In general, these device settings were largely very similar to those reported in the literature (63). The patients tended to respond at voltages that are much higher than those typically seen in patients with DBS for Parkinson’s Disease or essential tremor (average voltage 4.57, range 2.5 to 8.0 V). These high stimulation demands often led to a subset of the patients requiring battery changes as frequently as once every 6 months, and ultimately led to the majority (six of nine) of the patients undergoing implantation of rechargeable generators as discussed above. Pulse widths varied widely among patients, but in general larger pulse widths led to better response, with three of six responders programmed with pulse widths of 210 µs. The frequency used was 135Hz in all but one of the responders. Although our team trialed bipolar electrode settings in some of the patients, this was often poorly tolerated or ineffective; thus, 8 of 9 of the patients were noted to have their best response with unipolar settings, often with the deepest contacts—contact 0 or contact 1 for the negative pole—stimulated). This finding is consistent with previous literature suggesting stimulation on the border of the nucleus accumbens or in the ventral regions of the anterior limb of the internal capsule leads to the best response (33). Furthermore, our observation emphasizes the point that different brain regions likely require different stimulation parameters to induce therapeutic or neuromodulatory effects. The parameters used at one site cannot necessarily be extrapolated to a different brain target or region.

In general, patients were maintained on a single DBS program, which is in contrast to DBS as often employed for the treatment of movement disorders (64). Patients were rarely provided with multiple stimulation programs on their device or given control of their own device for adjustments, as this became a source of anxiety or rumination for some of the patients when trialed. This decision was often re-evaluated on a case-by-case basis, and a few of the patients were ultimately provided with the ability to adjust their device settings and take advantage of this additional flexibility without issue. The primary reason for developing multiple programs was to allow toggling between an efficacious setting and a more tolerable setting. For example, when one of the device settings led to greater therapeutic response but also had sleep-impairing side effects, a second more tolerable setting was incorporated for use prior to sleep.

One unique finding from our series was noted for patient 5, a right-handed female in her 60s with OCD and comorbid major depressive disorder, generalized anxiety disorder, and panic disorder. She responded to left unilateral DBS stimulation after multiple trials of right-sided stimulation produced symptoms of anxiety, restlessness, and panic. Even when the DBS system was turned on and off in a blinded fashion, the patient would experience these distressing symptoms with right-sided stimulation only, and the right VC/VS lead was subsequently turned off. Despite proceeding with only left unilateral DBS stimulation, patient 5 was able to achieve and maintain clinical benefit from her device. This observation is in contrast to previous speculation that OCD pathology may lateralize more to the right side of the brain and previous case reports demonstrating response to right unilateral DBS (65). While it is possible that our patient may have been a rare case of a right-handed woman who was right-brain dominant (and thus possibly more likely to respond to left unilateral DBS than the general population of OCD patients), to our knowledge this is the only case of a patient responding solely to left unilateral DBS of the VC/VS for OCD.

### Mechanism of Action

The question of the mechanism of action of DBS for OCD is a complicated and evolving theory that is reviewed elsewhere. In brief, it appears that DBS can modulate neural activity on several levels, including the inhibition of local neural firing, activation of orthodromic and antidromic axonal conduction, alteration of neurotransmitter levels, and “tuning” of neural firing patterns, information transmission, and inter-regional coherence (45). Some studies suggest that DBS can also alter neurogenesis (66). Although no explicit causal or mechanistic relationship can be derived from these data given the retrospective nature of our study, our findings can provide some useful and important insights on this topic.

One interesting observation from this review relates to the improvement in depressive symptomatology for the majority of OCD patients with comorbid depression. The VC/VS was our stimulation target in this patient population. This region is proximate to the nucleus accumbens, a structure commonly associated with reward processing, anhedonia, and motivation (67). Given these functions, it stands to reason that stimulation of VC/VS could have effects on mood and depression symptomatology in addition to OCD symptoms (68). Indeed, the nucleus accumbens and VC/VS targets have been the focus of DBS in previous clinical trials for treatment-resistant major depressive disorder (69, 70). Previous work has shown that stimulation at these targets can increase local and remote dopamine and 5-HT levels, and decrease the subgenual cingulate hyperactivity often associated with MDD (71–75). Although previous randomized controlled trials of VC/VS DBS in MDD have demonstrated mixed results, our results suggest that comorbid MDD may be a good prognostic indicator for OCD patients getting VC/VS DBS (70, 76). At the very least, a VC/VS target-of-choice should be strongly considered for OCD patients with comorbid MDD who are being evaluated for DBS. Additionally, deeper VC/VS targets providing stimulation near the nucleus accumbens may have a greater impact on relieving mood symptoms. These considerations raise the question of whether OCD with comorbid MDD represents a subtype of either illness, or a constellation of network pathology that is exquisitely responsive to VC/VS stimulation. We believe this specific question has not been investigated in a rigorous manner and could be a worthwhile focus of future research. It is possible OCD with comorbid MDD may be a different phenomenon with alternate targets of interest in contrast to MDD or OCD alone.

### Cost Analysis

To our knowledge, this report presents the first systematic tabulation and analysis of the hardware costs related to long-term use of a DBS system for OCD. We found that despite the higher initial hardware cost investment inherent to a rechargeable generator, the rechargeable device was more cost effective by the end of the second year of use when compared to a standard, non-rechargeable system (**Figure 3**). This cost savings occurs because the more energy-intensive device settings required by OCD patients leads to an average battery life of 1.4 years for a non-rechargeable battery. As a consequence of this shorter battery life, while we initially implanted our patients with non-rechargeable systems, all were later transitioned to rechargeable systems. Use of rechargeable impulse generators is an accepted preference in the treatment of movement disorders (77). Recently, de Vloo et al. reported their experience with the use of rechargeable impulse generators for OCD patients, in which they demonstrated feasibility for this patient population. While some provider concern exists about the development of obsessions surrounding charging and checking the battery (3), de Vloo reported that only two of their twelve patients showed some obsessions with charging the device; these obsessions were quickly remedied with scheduled daily charging and led to complete remission of the obsessive thoughts (40). We recommend that in the future, OCD DBS teams should strongly consider initially implanting a rechargeable system in patients for whom DBS is the treatment-of-choice.

### Limitations

Given the inherent limitations of a retrospective chart review, the data evaluated here was not obtained in a controlled setting but rather in a “real-world” clinical environment. It is promising to see similar outcomes in terms of response and remission rates among our sample compared to other centers (15, 38, 46). Given that these patients suffer from treatment-refractory OCD, on-going adjustments in medication and other therapeutic treatments, in addition to DBS adjustments, created some irregularity in follow-up and ongoing symptom assessment. This irregularity adds significant uncontrolled variability to the results, making measurement of the direct impact of the DBS itself (versus other psychopharmacologic, psychotherapeutic, and psychosocial factors) difficult. Of note, patients with OCD have lower placebo response rates compared to patients with other forms of anxiety disorders, and this population of patients with severely treatment-resistant OCD is likely to be on the extreme end of this lack of placebo effect (52). As such, the benefits of DBS described here are likely to be true effects.

As is common to most reports of DBS for OCD, our study included only a small number of patients. One recent review identified only 112 unique OCD patients mentioned in the literature with DBS implantation. The ability to generalize the effects of treatment is further hampered by the fact that these published cases include implantation at 6 different targets (78). Considering the almost-infinite parameter space available for adjusting and programming a DBS device, combined with the requisite and concurrent non-DBS treatment adjustments, it is no surprise that standardized guidelines on systematic programming strategies remain absent. We and others have documented the safety of DBS in severely impaired patients with limited alternate treatment options, and the collective data demonstrate that some patients experience remarkable and sometimes life-changing benefits. However, the small numbers of subjects and limited “real-world” or off-study outcome data have hampered the broad acceptance of DBS as a treatment modality for OCD. As such, it is of upmost importance that further studies be conducted to accumulate more experience and knowledge to inform future treatment decisions.

Another limitation inherent to retrospective studies is the lack of a standardized timeline for obtaining rating scales, and the variability in rating scales used to supplement YBOCS scores over the course of several years. Among the patients presented here, some patients had received a full battery of rating scales on a nearly monthly basis after implantation, whereas other patients have only a few rating scales obtained over the course of several years due to presumed stability of symptoms. This lack of consistently collected data makes it difficult to understand all of the acute or subacute changes in symptoms that might have been observed with more frequent follow-up. Indeed, this variability makes it impossible to capture the exact duration of response or remission of symptoms for some patients, thus introducing a source of potential error for calculation of response rate at any given time point. For example, at some follow-up visits patients were deemed clinically stable or improved by physician interview, and thus the clinician would forego obtaining a formal YBOCS score. In our analysis, we classified Patient 8 as a nonresponder as his last recorded YBOCS score demonstrated only 15% improvement. However, multiple subsequent clinic notes describe subjective improvement without a corresponding YBOCS score to quantify symptoms. This lack of YBOCS assessment was noted more commonly for patients who were stable or doing well clinically and thus may lead to a conservative underestimation of our treatment effect.

In regards to the variability in rating scales, the lack of consistently obtained non-OCD metrics made it difficult to draw conclusions related to non-OCD symptom domains—for example, depressive symptoms were variably measured using the Beck Depression Inventory; the HAMD-21 and 24; the QIDS16; the IDS30; and the PHQ9. Given this heterogeneity, it is difficult to draw conclusions beyond generalities about how a patient was functioning from visit to visit.

### Future Work

Future investigations should focus on controlled prospective studies with the aim of gaining further insights into the pathophysiology of OCD and the physiological changes induced by DBS. Thus far, the only studies of the physiology of VC/VS stimulation in living humans are on the level of case reports or case series. Nonetheless, the data described in this literature are intriguing and merit further scientific pursuit (10, 79). Larger studies of functional connectivity MRI or PET looking at changes in brain activity in OCD patients with DBS on and off could contribute significantly to providing insights about the OCD disease state and possible neuroimaging biomarkers to predict or better characterize response to DBS. An additional area of great need in the field lies in the development of guidelines for effective and efficient DBS programming. Most DBS programming protocols to date are based on work in the movement disorders field, where DBS is more frequently utilized for Parkinson’s Disease, essential tremor, and dystonia. Although helpful, these protocols were developed for different disease states associated with unique anatomic targets, and very little work has been done examining the optimal stimulation parameters for OCD targets, which are diverse and include the VC/VS, nucleus accumbens, inferior thalamic peduncle, and anterior limb of the internal capsule. Research focused on testing the parameter space of DBS for OCD at these targets is going to be essential for driving this field forward and promoting this valuable and promising technology for safe, appropriate, and responsible use on a wider scale. Considering that 2–3% of the population has OCD, and 10-40% of those patients are resistant to current therapeutic options, the number of patients who could potentially benefit from DBS dwarfs the number able to access and receive this treatment.

## Conclusions

This manuscript documents the development, implementation, and outcomes of a multidisciplinary program of DBS for OCD in a single academic hospital setting, with the intention of explicitly detailing our methods for others to replicate, critique, and build upon. We show that for some patients with severe OCD, DBS can be a viable and efficacious option. Additionally, we provide some guidance on optimizing device settings, noting that these patients typically require elevated voltages that consequently shorten battery life. Considering these high demands on the impulse generator, a cost analysis reveals that it would be more cost effective to initially implant a rechargeable device. Finally, we speculate on pathophysiologic mechanisms of OCD, the DBS effect on physiology, and predictors of treatment response, drawing upon examples in our patient cohort and comparing them to findings in other published literature.

OCD is a severe, disabling, and potentially fatal psychiatric illness, and new treatment modalities are desperately needed to address the symptoms of the large minority of sufferers who do not respond to medications and psychotherapy. DBS provides a versatile, safe and effective solution that requires diligent research and improved access for patients before it will reach its optimal therapeutic potential.

## Data Availability Statement

The datasets generated for this study are available on request to the corresponding author.

## Ethics Statement

The studies involving human participants were reviewed and approved by the University of Iowa Human Subjects Office. Written informed consent for participation in deep brain stimulation for obsessive compulsive disease was obtained from all participants at the implanting institution. Separate written informed consent was obtained from Patient 2 and Patient 4 for publication of their case vignettes.

## Author Contributions

MH, NT, LM, and JG prepared the manuscript draft with important edits and intellectual input from FJ, MZ, LC, and JB. All authors approved the final manuscript.

## Conflict of Interest

The authors declare that the research was conducted in the absence of any commercial or financial relationships that could be construed as a potential conflict of interest.
